# Awareness, attitude and perceived knowledge regarding First Aid in Kinshasa, Democratic Republic of Congo: A cross-sectional household survey

**DOI:** 10.1016/j.afjem.2022.03.001

**Published:** 2022-04-03

**Authors:** Ken Diango, John Yangongo, Vera Sistenich, Eric Mafuta, Lee Wallis

**Affiliations:** a Division of Emergency Medicine, Groote Schuur Hospital, University of Cape Town, Cape Town South Africa.; b Kinshasa School of Public Health, University of Kinshasa. Commune Lemba, Kinshasa, Democratic Republic of Congo; c Emergency Medicine Department, St George's Hospital, Gray Street, Kogarah, NSW, Australia

**Keywords:** First aid, emergency care, awareness, knowledge, Democratic Republic of Congo

## Abstract

**Introduction:**

Emergency care can potentially address half of deaths and one-third of disability in low-and-middle income countries. First Aid (FA) is at the core of out-of-hospital emergency care and is crucial to empower laypersons to preserve life, alleviate suffering and improve emergency response and outcomes. This study aimed to gauge FA awareness, the attitude and perceived knowledge in households in the low socioeconomic setting of Kinshasa, Democratic Republic of Congo (DRC).

**Methods:**

We undertook a cross-sectional community-based household survey in twelve health zones in Kinshasa. A three-stage randomised cluster sampling was used to identify 1217 households. The head of each household or an adult representative answered on behalf of himself/herself and the household. The primary outcome was FA awareness, attitude and perceived knowledge.

**Results:**

Most households had a poor socio-economic background, with 70.0% living on <US$100 per person per month. Most respondents received formal education (98.4%), with 37.6% reaching the tertiary level. The majority (77.6%) believed that an emergency requiring FA was likely to happen in their household. There was a noticeable contrast between awareness (90.0% asserted that FA knowledge is a necessity) and positive attitude regarding FA (91.3% believed that FA increases wellbeing and survival) on one hand, and the insignificant rate of FA training (0.2%) on the other. Most (83.6%) acknowledged they did not think they had the required basic FA knowledge and skills for five selected common life-threatening emergencies. The age, area of residence and level of education of participants played a variable role regarding FA awareness, attitude and knowledge.

**Conclusion:**

Most participants reported inadequate knowledge of FA despite awareness and a positive attitude. Context-appropriate training programs are greatly needed to empower Kinshasa's communities and equip them with lifesaving skills.

## African relevance

• Prehospital emergency care systems in Africa need to be developed to address a growing burden of disease and improve outcomes.
• Minimal data exist on First Aid (FA) in the low socioeconomic setting of Sub-Saharan Africa and the Democratic Republic of Congo (DRC) in particular.
• A community-based evaluation can provide a better understanding of the nature and level of the gaps as perceived by community members in Kinshasa, DRC.
• This evaluation offers a basis from which country-specific layperson FA training programmes could be developed and rolled out to equip Kinshasa's communities with lifesaving skills.

## Introduction

Emergency care can potentially address half of deaths and one-third of disability in low-and-middle income countries [1]. First aid (FA) is the immediate assistance provided to a sick or injured person until professional help arrives [2]. It is broader than basic life support and encompasses interventions seeking to preserve life, alleviate suffering, prevent further illness or injury, and promote recovery [2,3]. It is at the core of out-of-hospital emergency care (OHEC) and is crucial to improve emergency response and outcomes [3,4]. It is an essential first layer of care, especially in the context of inadequate access to prehospital care in low-income countries (LICs) [5]. Available data indicate significant mortality in LICs, particularly in sub-Saharan Africa, where a substantial proportion of deaths occur in the acute phase of illness or injury [6].Unfortunately, emergency care in this region is underdeveloped; less than one in three African countries has pre-hospital Emergency Medical Services in place [7]. Since most health emergencies occur far from trained personnel such as at home, schools, places of worship, sports fields, roadsides, etc, the community must play a key role in the initial management to influence outcomes and the overall impact of injuries and acute illnesses.

There is clear evidence regarding the benefits of FA at individual, household, community, regional and national levels [8,^9^,10,11,^12^]. Beyond saving lives and alleviating suffering, FA improves accident awareness and prevention by stimulating adherence to safety standards; it also enhances preparedness by encouraging practice of drills, elaboration of evacuation plans, and FA kit availability [13,14]. For the trained provider, it increases self-confidence and propensity to act when required [15]. Prevalent FA knowledge and practice can increase bystander cardiopulmonary resuscitation (CPR) and out-of-hospital cardiac arrest survival rates [16]. Advocates have called for the need to increase global access to FA by ensuring that at least one person in each household has access to FA training, regardless of their socioeconomic status or other potential discriminatory factors [17]. Poverty, low level of education, geographical accessibility, cost, prohibitive social norms and poor access to information are some of the reasons correlated with lack of awareness, poor access to, and nonacceptance of health services such as vaccination, contraception and cancer screenings in LICs [18,^19^]. These correlates also apply to community availability of FA.

Awareness and positive attitude are pre-requisite for FA knowledge and practice. Awareness is the state or ability to perceive or to be conscious of events, situations, facts, and can be self-reported or tested; it always implies information and knowledge [20]. Information on FA can be obtained from mass media, internet, social media, health professionals and bodies, special FA programmes and trainings [21]. Attitude can be defined as a negative or positive evaluation of a concept or object which influences behaviour towards it [22]. Knowledge is simply the state of being familiar with something, a concept or a topic; it essentially stems from learning and training and is anchored by practice [23]. FA knowledge and skills are essential for every adult and older child who is likely to be exposed one day to a life-threatening injury or acute illness [24] and can be useful in any setting.

Few studies have assessed layperson awareness, attitude, knowledge (perceived or objective) and practices regarding FA in the sub-Saharan Africa context [11,25,26,27,28,29]. There is currently no such baseline data for the Democratic Republic of Congo (DRC), a LIC with underdeveloped emergency care system and no formal ambulance services [30], and where one out of four people is at risk of dying prematurely [31]. As the country strives to develop its prehospital system, FA represents an opportunity to empower communities to contribute to a resilient OHEC system. In this context, it could be valuable to investigate layperson FA perceptions to understand the specific landscape and tailor adapted programmes to ensure higher prevalence of FA practice in the country. Increased FA knowledge and practice could potentially strengthen the community-based OHEC (Tier-1) by multiplying the number of community responders [32], which would subsequently impact on formal ambulance service (Tier-2) demand and utilisation, and ultimately improve access.

This community-based household survey about FA in DRC is aimed at gauging awareness, attitude and perceived knowledge regarding FA in households in the low socioeconomic setting of its capital city, Kinshasa.

## Methods

This study is part of a larger community-based cross-sectional household survey which aimed at evaluating the needs and supply of emergency care in Kinshasa and was conducted by a research team from the School of Public Health of the University of Kinshasa and the University of Cape Town.

The study was conducted in Kinshasa, the capital city of DRC. The DRC is located in Central Africa and has an estimated population around 90 million [33]. Its healthcare system is under resourced, underdeveloped and most health indicators are ‘concerning’ [30,31]. The challenges regarding optimal supply of healthcare are more pronounced regarding emergency care. There is no formal EMS system and no universal access number for the public to call in case of emergencies [30]. Kinshasa is home to almost 15 million people [34] and broadly representative of the country's demographic structure [33] .

Cluster sampling was used to calculate the requisite sample size. Convenience sampling was used to select twelve of the city's 35 health districts (HD) (zones de santé), three in each of the four city's administrative regions, based on data of previous comprehensive Data Health Survey [35]. A sample of 1060 households was generated for expected proportion with 5% absolute precision and 95% confidence. A 10% non-response rate was considered as conservative based on similar household surveys on emergency care in LIC [36,^37^]. Factoring this in, the study sampled 1217 households. Multiple socioeconomic features differentiate residents of more urban areas (UA – suburbs within a few kilometres of the Central Business District of Gombe) from those in peri-urban areas (PUA – less urbanised and more remote countryside). It was therefore worthwhile to segregate our sample population (UA - 1016 households from 10 HD and PUA - 201 households from 2 HD). A three-stage randomised cluster sampling was then used to identify the households, starting with the health areas (HA) (aires de santé) within the HD, followed by streets within HA, and lastly, households. In each household, the head of household or his/her representative was subjected to a comprehensive questionnaire on emergency care, with a section on first aid.

The survey tool used was included in the larger questionnaire on emergency care and adapted to the DRC context from similar studies on FA [9,12,25]. It encompassed 3 subsections: respondent demographics; previous experience with accidents, acute illness and death; FA awareness, attitude and perceived knowledge. A survey team of twelve experienced researchers fluent in the two majority languages of the area underwent a 2-day survey orientation for training, evaluation of competency in administering the protocol, and piloting of the survey for refinement. Over 10 days, surveyors worked in specific demarcated HA blocks during working hours each weekday plus one Saturday, starting at a convenient household, and then interviewing every 10th adjacent household until a sample of approximatively 100 households was reached. In the case where householders declined to participate or were all absent, surveyors moved to the next household immediately adjacent until they were able to conduct an interview, later resuming the systematic allocation from the original selection. Consenting household heads (or an adult representative) were asked a set of questions about emergency care, including FA. No survey responses were excluded.

The survey questionnaire was hosted on the SurveyCTO platform [38]. Responses were captured on password-protected tablets and uploaded daily on the secure server and10% of saved surveys responses were randomly checked daily by a researcher for adequacy and ongoing quality assurance. The raw data was securely downloaded and stored on a password-protected computer. Missing data points (unanswered questions) were left out from data entry. Chi-square testing and Fischer Exact testing were used to determine statistically significant differences between groups (two-sided significance level of p<0.01).

Ethics approvals were obtained from the School of Public Health of the University of Kinshasa (REF ESP/CE/077/2021) for the larger study this survey was part of.

## Results

In August 2021, 1217 households were surveyed, 1016 in urban areas (UA) and 201 in peri-urban areas (PUA) (Table 1). Respondents were predominantly female (68.1%), with a mean age of 39.9 ±14.4 years. Male respondents were mostly fathers in nuclear families (44%) while spouses represented 34.5% of participants. The majority were economically disadvantaged, living on <US$100 per person per month (70%). Poverty was higher in PUA than UA (39.0% versus 16.1% of households living on <US$100, p<0.01). There was a significantly higher proportion of respondents with tertiary level education in UA than in PUA (41.9% vs 14.9%, p<0.01)

**Table 1:** Respondent demographics.

	Urban areas		Peri urban areas		Total	
	n	%	n	%	n	%
	1016	83.5	201	16.5	1217	100
**Gender**						
Male	328	32.3	60	29.9	388	31.9
Female	688	67.7	141	70.1	829	68.1
**Age (years)**						
18-20	48	4.7	7	3.5	55	4.5
21-30	267	26.2	54	26.8	321	26.4
31-40	276	27.1	60	29.8	336	27.6
41-50	197	9.5	43	21.4	240	19.7
51-60	110	10.8	20	9.9	130	10.7
61-70	79	7.7	16	7.9	95	7.8
71+	39	3.8	1	0.5	40	3.3
Mean	40.1		39.0		39.9	
**Level of education**						
None	15	4.8	4	2.00	19	1.6
Primary	86	8.4	26	12.9	112	10.0
Secondary	489	48.1	141	70.1	630	51.8
Tertiary	426	41.9	30	14.9	456	37.6
**Employment**						
Unemployed	471	46.4	131	65.2	602	49.5
Public servants	177	17.4	18	8.9	195	16.0
Private sector	120	11.8	14	6.9	134	11.0
Self-employed	152	14.9	21	10.4	173	14.2
Others	96	9.4	17	8.4	113	9.3
**Total household income**						
< US$ 100	129	16.1	57	39.0	186	15.3
US$ 100-249	357	35.1	53	26.3	410	33.7
US$ 250-499	217	21.3	25	12.4	242	19.9
US$ 500-999	80	7.8	11	5.5	91	7.5
US$ 1000+	18	2.3	00	0.0	18	1.5
Couldn't say	215	21.1	55	27.3	270	22.2

Injuries or acute illnesses prompting emergency visits to a health facility were frequent (52.6%). Faced with an emergency requiring immediate intervention at home, 63.1% stated that they had no one in the neighbourhood to turn to for assistance. Ambulance use for emergency care and transportation was minimal (0.2%). A total of 155 deaths were reported in the twelve months prior to the study (12.8% of households), of which 20.6% occurred out-of-hospital (Table 2).

**Table 2:** Households experience with acute illness, accidents and death

	Urban areas		Peri urban areas		Total	
	n	%	n	%	n	%
	1016	83.5	201	16.5	1217	100
**Emergency visit of household members to a health facility in the last twelve months**						
	513	50.5	127	63.2	640	52.6
**Ambulance calls for emergencies in the last twelve months**						
	3	0.2	0	0.0	3	0.2
**Death in the household in last twelve months**						
In-hospital deaths	102	78.5	21	84.0	123	79.3
Out-of-hospital deaths	28	21.5	4	16.0	32	20.6
Total	130		25		155	12.7

There was a noticeable contrast between awareness and positive attitude regarding FA on one hand (90.0% confirmed that FA knowledge was a necessity and 91.3% believing FA helps improve outcomes), and the insignificant number of participants trained in FA on the other (0.2%) (table 3). In both settings and across all the ages groups, the majority (77.6%) believed that an emergency requiring FA was likely in their household. An average of 83.6% of participants acknowledged they didn't think they had the required basic FA knowledge and skills for five selected common life-threatening emergencies (choking, post-traumatic external haemorrhage, febrile seizure, obstructed airway in an unconscious adult and cardiac arrest).

**Table 3:** Awareness and attitude regarding FA.

	Urban areas areas		Peri urban		Total	
	n		n		n	%
	1016	83.5%	201	16.5%	1217	100%
**Believe an emergency requiring FA is likely to happen in household**					758	100
Total	461	78.4	126	21.4	588	77.6
No education	6	66.7	3	33.3	9	1.5
Primary + Secondary education	275	73.9	97	26.1	372	63.4
Tertiary education	180	87.4	26	12.6	206	35.1
**Believes FA knowledge is a necessity**					1184	100
Total	897	84.4	169	15.6	1066	90.0
No education	14	82.4	3	17.6	17	1.6
Primary + Secondary education	505	78.3	140	21.7	645	60.5
Tertiary education	378	93.6	26	6.4	404	37.9
**Has undergone a formal FA training**					1217	100
Total	3	100	0	0.0	3	0.25
**Possibility for a neighbour to assist with FA measures**					1217	100
Total	344	33.9	105	52.2	449	36.9
**Previously faced an emergency requiring FA**					1172	100
Total	511	92.4	42	7.6	553	47.2
Felt FA was urgently needed	340	95.0	18	5.0	358	64.7
Felt confident he/she knew to apply FA measures	198	94.7	11	5.3	209	37.9
Felt FA training would have made him/her more comfortable	461	92.7	36	7.3	497	89.9
**Never faced emergency requiring FA but thinks FA helps improve outcomes**					587	100
	415	77.4	121	22.6	536	91.3

While respondents with tertiary education represented 37.6% of our study population, they accounted for 64.3% of participants with self-perceived FA knowledge (p<0.01). This wasn't the case respectively with FA awareness (for which 35.1% were respondents with higher education) and positive attitude (for which the highly educated accounted for 37.9%) (table 4). Residents of UA represented 83.6% of our sample, but 91.0% of participants with self-perceived FA knowledge lived in UA (p<0.01), likely due the higher proportion of residents with tertiary education in UA versus PUA (41.9% vs 14.9%, p<0.01). However, area of residence did not appear to make a significant difference regarding FA awareness (UA residents accounted for 84.4% while they represented 83.2% of the study population) and positive attitude (UA residents accounted for 77.4%). Lastly, while respondents aged <31years represented 30.9% of our study population, they accounted for 25.0% of participants with self-reported FA knowledge. It was the opposite with the ≥51years (21.8% of total population; 23.9% of those with self-reported FA knowledge). Though non-significant, this trend may suggest that younger respondents felt less knowledgeable than the older. Age was a significant discriminator regarding awareness (20.2% for <31years and 31.3% for ≥51years, p<0.01) and positive attitude (20.8% for <31years and 31.4% for ≥51years, p<0.01).

**Table 4:** Respondents perceived knowledge regarding FA.

	Urban areas		Peri urban areas		Total	
	n		n		n	%
	1016	83.5%	201	16.5%	1217	100%
**Believes he/she has the FA knowledge for:**						
	**1) 5-year-old child in respiratory distress due to chocking**				1172	100
Total	153	85.0	27	15.0	180	15.4
No education	1		2		3	1.7
Primary + secondary	52		22		74	41.1
Tertiary education	100		3		103	57.2
	**2) 12-year-old with an open broken leg bleeding profusely**				1172	100
Total	252	91.6	23	9.4	275	23.5
No education	2		1		3	1.1
Primary + secondary	85		18		103	37.4
Tertiary education	165		4		169	61.4
	**3) Adult in cardiopulmonary arrest**				1170	100
Total	111	95.5	4	4.5	115	9.8
No education	0		0		0	0.0
Primary + secondary	30		2		32	27.8
Tertiary education	81		2		83	72.2
	**4) 2-year-old infant with febrile seizures**				1170	100
Total	281	92.1	24	7.9	305	26.1
No education	0		0		0	0.0
Primary + secondary	151		19		170	55.7
Tertiary education	130		5		135	44.3
	**5) Adult found unconscious with noisy breathing**				1171	100
Total	81	92.0	7	8.0	88	7.5
No education	0		0		0	0.0
Primary + secondary	8		4		12	13.6
Tertiary education	73		3		76	86.3
					1171	100
**Average knowledge**	**175**	**91.0**	**17**	**9.0**	**192**	**16.4**

## Discussion

This study gauged FA awareness, attitude and perceived knowledge in households of Kinshasa. Our 1217 respondents were mostly female (68.1%), had a mean age of 39.9 ± 14.4 years, and had a variability which allowed an assessment of probable differences in awareness and perceptions based on the age [39]. Most households were economically disadvantaged, with 70.0% living on <US$100 per person per month and unemployment rate of 49.5%. Households in UA differed significantly from those in PUA by their respondents’ higher level of education and lesser unemployment rates, and this likely affected their average income. Our data suggest that these differences may have affected these subgroups perceptions and knowledge of FA. As it is the case for the lack of awareness, poor knowledge and non-acceptance of vaccination and contraception in LICs, poverty, low level of education and poor access are among factors negatively affecting FA prevalence in communities [40]. Health emergencies, broadly defined as illnesses or injuries requiring medical care within few minutes or hours, were a common occurrence in households, with emergency visits in the twelve months prior to the survey recorded in 52.6% households, congruent with other LICs studies [21,26]. Some of these acute presentations could require FA. In contrast, utilisation of ambulance services for emergency care was almost non-existent (0.2%), far below other African countries (South Africa-67%, Ethiopia-20.3% and Ghana-4.5%) [37,41,^42^]. Nevertheless, even in well-resourced countries with efficient EMS, when faced with emergencies, household members still play a critical role in promptly activating the response and applying FA, making FA an essential link in the chain of care in any setting [3,4].. Laypersons without FA training are left with the unrealistic option of getting a medically trained or more knowledgeable neighbour to assist (not a possibility in 63.1% in our study). Of the 155 deaths which occurred in the households in the twelve months prior to our study, 20.6% were out-of-hospital. FA by a layperson was the only option of care in those circumstances and could have prevented some deaths. A systematic review on first aid provided by laypeople to trauma victims showed a potential mortality reduction if first aid is administered [43].

Most participants (77.6%) agreed that an emergency requiring FA was likely to occur some day in their household, and 90.0% acknowledged that FA knowledge was a necessity. This speaks to the awareness of most respondents of the value of FA. Though our study did not specifically investigate participants sources of FA knowledge, the non-significant difference of awareness noted between PUA and UA groups may suggest a lesser availability of sources in the first subgroup. Despites heightened awareness, FA training in Kinshasa was minimal and significantly lower than in comparable LIC settings [9,25] and high-income countries [8,12]. This is likely multifactorial, including scarcity of trainings available rather than a lack of interest. There are limited and sporadic FA training opportunities run mainly by the Red-Cross, mostly in cities, and often to build capacity among selected community workers [44]; the country's official primary and secondary schools’ curricula do not specifically list FA skills training as a subject [45].

Furthermore, our data reinforce the vital need for practical FA training in Kinshasa; respondents’ lived experience demonstrate the lack of FA practice in households. Regardless of their residential area, a non-negligible proportion of respondents (47.2%) stated that they themselves had directly faced an emergency requiring FA in the past, and 62.1% of those lacked self-confidence in their ability to administer FA. Experience of unintentional injuries were found to be one of the predictive factors for FA awareness and knowledge [46]. Congruent with the literature [13,14,^47^], 90% of these respondents believed, regardless of their level of education, that training would have made them more confident. Training, particularly recent exposure, has been associated with higher perceived FA skills and increased expected and actual application of those skills [48]. Even among those who never faced an emergency requiring FA, 91.3% believed that formal training can indeed help improve outcomes [3,4,^12^]. This positive attitude towards FA has been found in many similar studies [10,25,^48^].

An average 83.6% participants declared that they did not think they had the FA knowledge for five common emergencies scenarios, which corresponds to a knowledge rate inferior to most similar studies [12,18,^25^]. This lack of FA knowledge correlated with lack of FA in practice. In fact, knowledge essentially stems from learning and training [23]. Most respondents were unfamiliar with basic FA techniques such as back blows and abdominal thrusts for a choking child, haemorrhage control by direct pressure, airway opening and lateral recovery position for the unconscious breathing person and chest compressions for cardiac arrest. In this regard, the accepted, objective and accurate way to gauge knowledge (theoretical and practical) is by formal methodical assessment. However, self-declared lack of knowledge can be an indirect indicator [49,^50^].It is likely that some of the few who claimed to know FA techniques for the five scenarios in our study do not actually master them. Additionally, our data showed clear association between self-perceived knowledge and the level of education. There was greater proportion of participants with tertiary education among those with self-perceived FA knowledge compared to respondents with lower education. This is consistent with the literature confirming that education is strongly correlated with determinants of health and influences heath perceptions and practices [18]. Similarly, there were patterns also suggesting some level of association between older age and greater awareness, positive attitude and better knowledge, in keeping with findings of a study that investigated teachers in Ethiopia [25]. These factors are worth investigating further.

This research is based on self-reported data and the accuracy of responses provided by respondents could not be independently confirmed. Furthermore, some participants may have had recall bias or provided socially desirable answers. Additionally, terms like “first aid”, “health emergencies” or “knowledge” used in the questionnaire have broad definitions and, despite surveyors attempts to clearly explain meanings, could have been understood differently. Finally, a more accurate way to assess FA knowledge would have been to objectively and practically test and grade it instead of self-declaration. However, this foundational study offers the basis from which more comprehensive research on FA can be done in the future.

First aid knowledge is a vital life skill for every adult and older child. Despite awareness and a positive attitude, there is currently inadequate knowledge of FA in households in Kinshasa, DRC. Context-appropriate training programs are greatly needed to empower communities to preserve life, alleviate suffering and improve emergency response as well as outcomes. As part of the efforts to strengthen the prehospital care system, it is essential to strive to provide appropriate FA training programs to as many people as possible in Kinshasa.

## Dissemination of Results

A French translation of this article can be found in Appendix A. The findings of this study will be collated into reports for the DRC Ministry of Health to help inform FA programmes planning and development. They will also be written into short-format manuscript for publication in local web-based platforms and presented in various academic forums and advocacy for the community health organisations. They will be further discussed at the forthcoming African Conference on Emergency Medicine.

## Authors’ contribution

Authors contributed as follow to the conception or design of the work; the acquisition, analysis, or interpretation of data for the work; and drafting the work or revising it critically for important intellectual content: KD contributed 50%, LW 20%, EM 15%, VS 10% and JY 5%. All authors approved the version to be published and agreed to be accountable for all aspects of the work.

## Funding

The larger study this survey was part of was funded by a grant from the United Kingdom Royal College of Emergency Medicine (Reference RCEM LICG/2019/1)

## Declaration of Competing Interest

The authors declared no conflicts of interest

## Appendix Supplementary materials

Image, application 1
