# Supplementary material for: Awareness, attitude and perceived knowledge regarding First Aid in Kinshasa, Democratic Republic of Congo: A cross-sectional household survey
Source: Afr J Emerg Med. 2022 Apr 3;12(2):135–40. doi: 10.1016/j.afjem.2022.03.001 (PMC8980329; doi:10.1016/j.afjem.2022.03.001)
Supplement: Supplementary file 1 [file mmc1.docx]

**Perceptions, attitudes et connaissances sur les premiers secours**

**à Kinshasa, République Démocratique du Congo :**

**Une enquête transversale auprès des ménages.**

***Ken Diango*^1^*, John Yangongo*^2^*, Vera Sistenich, Eric Mafuta*^2^*, Lee Wallis*^1^**

^1^*Division de Médecine d'Urgence, F51-62, Ancien Bâtiment Principal, Hôpital Groote Schuur,*

*Faculté des Sciences de la Santé, Université du Cap, Observatory, Le Cap, 7925, Afrique du Sud.*

^2^ *Ecole de Santé Publique de Kinshasa, Université de Kinshasa. Commune de Lemba,*

*Kinshasa, République Démocratique du Congo*

^3^*Service de Médecine d'urgence, Hôpital St George, Gray Street, Kogarah NSW 2217, Australie.*

Introduction : Les soins d'urgence peuvent influer sur la moitié des décès et le tiers des invalidités qui surviennent dans les pays à revenu faible ou intermédiaire. Les premiers secours (PS) sont en première ligne des soins d'urgence préhospitaliers ; ils sont essentiels pour doter les membres de la communauté de la capacité de préserver la vie, soulager la souffrance et améliorer la riposte face aux urgences. Cette étude a pour but d’évaluer les perceptions, attitudes et connaissances sur les premiers secours au sein des ménages de Kinshasa, en République Démocratique du Congo.

Méthodologie : Une enquête transversale a été menée auprès de 1217 ménages de Kinshasa identifiés suivant un échantillonnage aléatoire en grappes à trois degrés. Le chef de ménage ou un représentant adulte a répondu en son nom propre et pour tout le ménage.

Résultats : La majorité des ménages étaient économiquement défavorisés (70,0% vivant avec moins de 100USD par personne par mois). Les femmes étaient majoritaires (68,1%) et 37,6% des répondants avaient le niveau éducation tertiaire. Il y avait un contraste entre la reconnaissance de l’importance des PS (90,0% ayant affirmé que leur connaissance est une nécessité) et une attitude positive d'une part (91,3% ayant déclaré que les PS accroissent le bien-être et les chances de survie), et le taux insignifiant des répondants formés en PS d'autre part (0,3%). La plupart des participants (83,5%) ont reconnu ne pas connaitre les PS à administrer dans cinq urgences courantes choisies. L'âge, le lieu de résidence et le niveau d'éducation des participants ont diversement influencé les perceptions, attitudes et connaissances des répondants sur les PS.

Conclusion : La majorité des participants ont démontré une connaissance insuffisante des premiers secours, malgré une reconnaissance de leur importance et une attitude positive. Des programmes de formation adaptés sont indispensables pour doter les communautés de Kinshasa de compétences essentielles en secourisme.

Mots clés : Premiers secours, soins d'urgence, perceptions, connaissance subjective, RD Congo.

**INTRODUCTION**

Les soins d'urgence ont le potentiel d’influer sur la moitié des décès et le tiers des invalidités qui surviennent dans les pays à revenu faible ou intermédiaire^[[1]](#endnote-1)^. Les premiers secours (PS) représentent l'assistance immédiate fournie à une personne malade ou blessée en attendant l'arrivée des secours professionnels^[[2]](#endnote-2)^. Ils ont un sens plus large que les soins de réanimation de base et comprennent des interventions visant à préserver la vie, soulager la souffrance, prévenir l’aggravation des maladies et blessures, et favoriser le rétablissement d’une victime^2^ ^[[3]](#endnote-3)^. Ils sont fondamentaux aux soins d'urgence préhospitaliers et améliorent le pronostic vital^3^ ^[[4]](#endnote-4)^. Les PS constituent aussi la première étape de la chaine des soins de santé, en particulier dans le contexte d'accès limité aux soins préhospitaliers dans les pays à faible revenu (PFR)^[[5]](#endnote-5)^. Les données disponibles indiquent une mortalité accrue dans ces pays, particulièrement en Afrique subsaharienne où beaucoup de décès surviennent dans la phase aiguë des maladies ou blessures^[[6]](#endnote-6)^. Malheureusement, les systèmes de soins préhospitaliers y sont peu développés ; moins d'un pays africain sur trois dispose de service d’aide médicale urgente (SAMU)^[[7]](#endnote-7)^. Étant donné que la plupart des urgences médicales surviennent loin des prestataires qualifiés, à la maison, dans les écoles, les lieux de culte, les terrains de sport, au bord de la route, la communauté est appelée à jouer un rôle crucial dans la prise en charge initiale et influer ainsi sur leur impact global.

Plusieurs données probantes démontrent les avantages des PS au niveau tant individuel que familial, communautaire, régional et national^[[8]](#endnote-8)^ ^[[9]](#endnote-9)^ ^[[10]](#endnote-10)^ ^[[11]](#endnote-11)^ ^[[12]](#endnote-12)^. En plus de sauver des vies et atténuer la souffrance, les PS améliorent la reconnaissance des risques et la prévention des accidents en incitant au respect des normes de sécurité ; ils améliorent également la préparation aux urgences en encourageant la pratique d’exercices de simulation, l'élaboration de plans d'évacuation et la disponibilité des kits de premiers secours^[[13]](#endnote-13)^ ^[[14]](#endnote-14)^. Pour le prestataire formé, la maitrise des gestes des PS augmente la confiance en soi et la propension à intervenir quand c’est nécessaire^[[15]](#endnote-15)^. Une connaissance accrue des PS au sein d’une communauté augmente la pratique de la réanimation cardiopulmonaire (RCP) et le taux de survie après arrêt cardiaque^[[16]](#endnote-16)^. Des plaidoyers ont ainsi appelé à accroitre l'accès global aux PS de telle sorte qu'au moins une personne dans chaque ménage ait accès à la formation aux PS, indépendamment de son statut socio-économique ou d'autres facteurs discriminatoires^[[17]](#endnote-17)^. La pauvreté, un bas niveau d'éducation, l'accessibilité géographique, le coût, les normes sociales prohibitives et le manque d'accès aux sources d'information sont quelques facteurs liés à l’ignorance, au manque d'accès et à la non-acceptation des services de santé tels que la vaccination, la contraception et le dépistage du cancer dans les PFR^[[18]](#endnote-18)^ ^[[19]](#endnote-19)^. Ceci est aussi vrai pour les PS au sein des communautés. Reconnaitre l’importance des PS et avoir une attitude positive à leur égard sont des prérequis pour leurs connaissance et pratique. Cette prise de conscience implique inévitablement un accès à l’information sur le sujet et l’acquisition du savoir^[[20]](#endnote-20)^. Les informations sur les PS peuvent être obtenues auprès des médias, sur internet, dans les réseaux sociaux, auprès des professionnels et associations de santé et divers programmes spéciaux^[[21]](#endnote-21)^. L'attitude peut être définie comme une évaluation positive ou négative d'un concept, d’une personne ou d'un objet qui influence le comportement à son égard^[[22]](#endnote-22)^. La connaissance quant à elle est simplement le fait d’être familier avec un concept, un sujet ou un objet ; elle découle essentiellement de l'apprentissage et de la formation, et est ancrée par la pratique^[[23]](#endnote-23)^. Les connaissance et compétences en PS sont essentielles pour tout adulte ou grand enfant susceptible d'être exposé un jour à un accident grave ou à une maladie aiguë^[[24]](#endnote-24)^.

Quelques études ont évalué les perceptions, attitudes, connaissances (objectives ou subjectives) et la pratique des PS en Afrique subsaharienne^11^ ^[[25]](#endnote-25)^ ^[[26]](#endnote-26)^ ^[[27]](#endnote-27)^ ^[[28]](#endnote-28)^ ^[[29]](#endnote-29)^. Il n'existe cependant pas de données de référence pour la République Démocratique du Congo (RDC), un PFR dont plusieurs indicateurs de santé sont préoccupants^[[30]](#endnote-30)^, et dont le système de soins d'urgence est sous-développé et dépourvu de SAMU^[[31]](#endnote-31)^. Vu que le pays s'est récemment engagé sur la voie de la Couverture Santé Universelle, les PS représentent une opportunité de responsabiliser et outiller les membres de la communauté afin qu’ils contribuent au développement d’un système préhospitalier résilient. Dans ce contexte, il serait utile d'enquêter sur les perceptions des membres de la communauté sur les PS pour comprendre les particularités locales et adapter des programmes de formation afin de disséminer la pratique des PS à travers le pays. Une connaissance et une pratique accrues des PS ont en effet le potentiel de renforcer les soins d’urgences préhospitaliers communautaires^[[32]](#endnote-32)^ en multipliant le nombre de secouristes potentiels, ce qui aurait à son tour un impact sur la demande et l'utilisation du SAMU, et améliorerait en définitive l'accès aux soins de santé.

Cette enquête auprès des ménages a pour objectif d’évaluer les perceptions, attitudes et connaissances concernant les PS dans les ménages de Kinshasa, la capitale de la RDC.

**METHODOLOGIE**

Cette étude fait partie d'une plus large enquête transversale auprès des ménages visant à évaluer les besoins et l'offre de soins d'urgence à Kinshasa, enquête menée par une équipe de recherche de l'École de Santé Publique de l'Université de Kinshasa et de l'Université du Cap.

L'étude a été menée à Kinshasa, la capitale de la RDC. La RDC est un pays d’Afrique centrale avec une population estimée à environ 90 millions d'habitants^[[33]](#endnote-33)^. L'état général des soins de santé y demeure préoccupant, avec plusieurs indicateurs anormaux^30 31^. Les défis relatifs à l'offre optimale de soins de santé primaires sont plus prononcés en ce qui concerne les soins d'urgence ; il n'existe pas de SAMU intégré et pas de numéro vert unique pour appeler les secours. Avec près de 15 millions d’habitants^[[34]](#endnote-34)^, Kinshasa est une ville-province dont le profil démographique est semblable au reste du pays^33^ .

L'échantillonnage en grappes a été utilisé pour calculer la taille d'échantillon requise. Un échantillonnage de commodité a été utilisé pour sélectionner 12 des 35 zones de santé (ZS) que compte la ville, en suivant les données d’une précédente large enquête (Data Health Survey)^[[35]](#endnote-35)^. Un échantillon de 1060 ménages a été généré pour la proportion attendue avec une précision absolue de 5% et un intervalle de confiance de 95%. Un taux de non-réponse de 10 % a été projeté sur la base d'enquêtes de ménages similaires sur les soins d'urgence dans les PRF^[[36]](#endnote-36)^ ^[[37]](#endnote-37)^. L'étude a ainsi échantillonné 1217 ménages. Il ressort de l’étude DHS^35^ que plusieurs caractéristiques socio-économiques différencient les habitants des zones urbaines (ZU – banlieues à quelques kilomètres du centre des affaires de la Gombe) de ceux des zones périurbaines (ZPU – quartiers moins urbanisés et plus reculés). Il était donc logique de distinguer au sein de notre échantillon une population en ZU (1016 ménages issus de 10 ZS) et une population en ZPU (201 ménages issus de 2 ZS). Un échantillonnage aléatoire en grappes à trois degrés a ensuite permis d’identifier les ménages, en commençant par les aires de santé (AS) au sein des ZS, suivies par les rues dans les AS, et enfin, les ménages. Dans chaque ménage, le chef de ménage ou son représentant a été soumis à un questionnaire détaillé sur les soins d'urgence, avec une partie sur les premiers secours.

**Protocole d'enquête**

L'outil d'enquête utilisé a été élaboré à partir d'études similaires sur les PS^9 12 25^, adapté au contexte de la RDC et inclus dans le large questionnaire sur les besoins et l’offre des soins d'urgence à Kinshasa. Il comprenait 3 parties : données sociodémographiques des répondants ; expérience antérieure d'accidents, maladies aiguës et décès ; perceptions, attitudes et connaissances sur les PS. Une équipe de 12 chercheurs expérimentés parlant couramment les deux langues majoritaires dans la ville (Français et Lingala) a reçu une formation de 2 jours pour s’imprégner des détails de l'enquête, faire une évaluation des compétences dans l'administration du questionnaire et des exercices pour son perfectionnement. Pendant 10 jours, les enquêteurs ont parcouru des blocs bien délimités dans les AS pendant les heures de travail chaque jour de la semaine plus un samedi, en commençant par un ménage convenable, puis en interrogeant le ménage à chaque dixième domicile, jusqu’à ce qu'un échantillon d'environ 100 ménages soit atteint. Dans le cas où un ménage refusait de participer ou personne n’était présent, les enquêteurs se déplaçaient vers le ménage au domicile immédiatement adjacent jusqu'à ce qu'ils soient en mesure de mener une interview, après quoi ils reprenaient l'attribution systématique de la sélection initiale. Le chef de ménage (ou un représentant adulte) ayant consenti à l’étude a répondu à une série de questions sur les soins d'urgence, y compris les PS. Aucune réponse n'a été exclue.

Le questionnaire d'enquête était hébergé sur la plateforme SurveyCTO^[[38]](#endnote-38)^. Les réponses étaient saisies sur des tablettes avec mot de passe et uploadées quotidiennement. Environs 10 % des réponses enregistrées étaient vérifiées quotidiennement de façon aléatoire par un chercheur pour s’assurer de la qualité. A la fin, les données brutes ont été téléchargées et gardées sur un ordinateur avec mot de passe. Les points de données manquants (questions spécifiques sans réponses) ont été exclus de la saisie des données. Le test du chi carré et le test Fischer Exact ont été utilisés pour déterminer les différences entre les groupes (seuil de signification p<0,01).

L’étude sur les besoins et l’offre de soins d’urgence à Kinshasa dont cette étude est une partie a obtenu l’approbation du Comité d’Ethique de l'Ecole de Santé Publique de l’Université de Kinshasa (REF ESP/CE/077/2021). En outre, des autorisations administratives ont été obtenues auprès du Gouvernorat de la ville de Kinshasa et des autorités des zones de santé choisies.

**RESULTATS**

| Tableau 1. Données sociodémographiques | | | | | | |
| --- | --- | --- | --- | --- | --- | --- |
|  | **Zones urbaines Zones périurbaines** | | | | **Total** | |
|  | **n** | **%** | **n** | **%** | **n** | **%** |
|  | **1016** | **83.5** | **201** | **16.5** | **1217** | **100** |
| Sexe | | | | | | |
| Homme | 328 | 32.3 | 60 | 29.9 | 388 | 31.9 |
| Femme | 688 | 67.7 | 141 | 70.1 | 829 | 68.1 |
| Age (années) | | | | | | |
| 18-20 | 48 | 4.7 | 7 | 3.5 | 55 | 4.5 |
| 21-30 | 267 | 26.2 | 54 | 26.8 | 321 | 26.4 |
| 31-40 | 276 | 27.1 | 60 | 29.8 | 336 | 27.6 |
| 41-50 | 197 | 9.5 | 43 | 21.4 | 240 | 19.7 |
| 51-60 | 110 | 10.8 | 20 | 9.9 | 130 | 10.7 |
| 61-70 | 79 | 7.7 | 16 | 7.9 | 95 | 7.8 |
| 71+ | 39 | 3.8 | 1 | 0.5 | 40 | 3.3 |
| Mean | 40.1 |  | 39.0 |  | 39.9 |  |
| Niveau d’éducation | | | | | | |
| Jamais scolarisé | 15 | 4.8 | 4 | 2.00 | 19 | 1.6 |
| Primaire | 86 | 8.4 | 26 | 12.9 | 112 | 10.0 |
| Secondaire | 489 | 48.1 | 141 | 70.1 | 630 | 51.8 |
| Tertiaire | 426 | 41.9 | 30 | 14.9 | 456 | 37.6 |
| Emploi | | | | | | |
| Sans emploi | 471 | 46.4 | 131 | 65.2 | 602 | 49.5 |
| Fonctionnaire | 177 | 17.4 | 18 | 8.9 | 195 | 16.0 |
| Secteur privé | 120 | 11.8 | 14 | 6.9 | 134 | 11.0 |
| Indépendant  Autre | 152  96 | 14.9  9.4 | 21  17 | 10.4  8.4 | 173  113 | 14.2  9.3 |
| Revenu total du ménage | | | | | | |
| < US$ 100 | 129 | 16.1 | 57 | 39.0 | 186 | 15.3 |
| US$ 100-249 | 357 | 35.1 | 53 | 26.3 | 410 | 33.7 |
| US$ 250-499 | 217 | 21.3 | 25 | 12.4 | 242 | 19.9 |
| US$ 500-999 | 80 | 7.8 | 11 | 5.5 | 91 | 7.5 |
| US$ 1000+ | 18 | 2.3 | 00 | 0.0 | 18 | 1.5 |
| N’a pas dit | 215 | 21.1 | 55 | 27.3 | 270 | 22.2 |
|  |  |  |  |  |  |  |

En Août 2021, 1217 ménages ont été interviewés, 1016 dans les zones urbaines (ZU) et 201 dans les zones périurbaines (ZPU) (Tableau 1). Les répondants étaient majoritairement des femmes (68,1%) et l’âge moyen était de 39,9 ± 14,4 ans. Il s’agissait principalement de pères de familles nucléaires ou leurs conjointes (78,5%). La majorité (70,0%) étaient économiquement défavorisés, vivant avec <100USD par personne par mois. La pauvreté était plus profonde dans les ZPU que dans les ZU (39,0% contre 16,1% des ménages vivant avec < 100USD, p<0,01). Le niveau d’éducation était plus élevé en ZU comparé aux ZPU (41,9% contre 14,9% avec niveau supérieur, p<0,01)

| Tableau 2. Expérience antérieure d'accidents, maladies aiguës et décès | | | | | | | | | | |
| --- | --- | --- | --- | --- | --- | --- | --- | --- | --- | --- |
|  | **Zones urbaines Zones périurbaines** | | | | | | | **Total** | | |
|  | **n** | **%** | | **n** | **%** | | | **n** | | **%** |
|  | **1016** | **83.5** | | **201** | **16.5** | | | **1217** | | **100** |
|  |  |  | |  |  | | |  | |  |
|  |  |  |  | | |  |  | |  | |
| Consultation en urgence d’un membre du ménage au cours des 12 derniers mois | | | | | | | | | | |
|  | 513 | 50.5 | 127 | | | 63.2 |  | | 640 | 52.6 |
| Appels d'ambulance pour les urgences au cours des 12 derniers mois | | | | | | | | | | |
|  | 3 | 0.2 | | 0 | | 0.0 |  | | 3 | 0.2 |
| Décès dans le ménage au cours des 12 derniers mois | | | | | | | | | | |
| Ds formation sanitaire | 102 | 78.5 | | 21 | | 84.0 |  | | 123 | 79.3 |
| En dehors de Fo.Sa. | 28 | 21.5 | | 4 | | 16.0 |  | | 32 | 20.6 |
| Total | 130 |  | | 25 | |  |  | | 155 | 12.7 |
|  |  |  | |  | |  |  | |  |  |

| Tableau 3. Perceptions et attitude concernant les premiers secours (PS) | | | | | | |
| --- | --- | --- | --- | --- | --- | --- |
|  | **Zones urbaines Zones périurbaines** | | | | **Total** | |
|  | **n** |  | **n** |  | **n** | **%** |
|  | **1016** | **83.5%** | **201** | **16.5%** | **1217** | **100%** |
| Croit qu'une urgence nécessitant les PS est probable dans le ménage 758 100 | | | | | | |
| Total | 461 | 78.4 | 126 | 21.4 | 588 | 77.6 |
| Jamais scolarisé | 6 | 66.7 | 3 | 33.3 | 9 | 1.5 |
| Niveau Primaire + Secondaire | 275 | 73.9 | 97 | 26.1 | 372 | 63.4 |
| Niveau Tertiaire | 180 | 87.4 | 26 | 12.6 | 206 | 35.1 |
| Croit que la connaissance des PS est une nécessité 1184 100 | | | | | | |
| Total | 897 | 84.4 | 169 | 15.6 | 1066 | 90.0 |
| Jamais scolarisé | 14 | 82.4 | 3 | 17.6 | 17 | 1.6 |
| Niveau Primaire + Secondaire | 505 | 78.3 | 140 | 21.7 | 645 | 60.5 |
| Niveau Tertiaire | 378 | 93.6 | 26 | 6.4 | 404 | 37.9 |
| A reçu une formation en PS 1217 100 | | | | | | |
| Total | 3 | 100 | 0 | 0.0 | 3 | 0.25 |
| Possibilité pour un voisin d'aider à administrer les PS 1217 100 | | | | | | |
| Total | 344 | 33.9 | 105 | 52.2 | 449 | 36.9 |
| A été confronté dans le passé à une urgence nécessitant des PS 1172 100 | | | | | | |
| Total | 511 | 92.4 | 42 | 7.6 | 553 | 47.2 |
| A estimé que les PS étaient | | | | | | |
| nécessaires de toute urgence | 340 | 95.0 | 18 | 5.0 | 358 | 64.7 |
| S’est senti confiant de son habilité | | | | | | |
| à administrer les PS | 198 | 94.7 | 11 | 5.3 | 209 | 37.9 |
| A cru qu’une formation en PS | | | | | | |
| l’aurait rendu plus confortable | 461 | 92.7 | 36 | 7.3 | 497 | 89.9 |
| Pense que les PS aide à améliorer le pronostic 587 100 | | | | | | |
|  | 415 | 77.4 | 121 | 22.6 | 536 | 91.3 |

**Expériences antérieures d'accidents, de maladies aiguës et de décès**

Les blessures et maladies aiguës ayant résulté en des consultations en urgence étaient fréquentes (52,6%). Face à une urgence nécessitant une intervention immédiate à domicile, 63,1 % ont déclaré n'avoir personne dans le voisinage vers qui se tourner pour une assistance. L'utilisation d’ambulances était insignifiante (0,2%). Au total, 155 décès ont été enregistrés au cours des 12 mois précédant l'étude (12,8% des ménages), dont 20,6 % survenus hors d’un établissement de santé (43,8% à domicile et 43,8% en route vers un établissement de santé).

**Perceptions, attitudes et connaissances subjectives sur les PS**

Il y avait un contraste éloquent entre la reconnaissance de l’importance des PS et une attitude positive d'une part (90,0% ont affirmé que la connaissance des PS est une nécessité et 91,3% que les PS aident à améliorer les résultats), et le nombre insignifiant de participants formés aux PS d'autre part (0,3%). Indépendamment du milieu et du groupe d'âge, la majorité (77,6%) a estimé probable la survenue un jour dans leurs ménages d'une urgence nécessitant des PS. Parmi les 47,7% des répondants qui ont déclaré avoir déjà eux-mêmes fait face à une urgence nécessitant des PS dans le passé, seuls 46,4% s’étaient senti confiants de posséder les connaissances nécessaires ; neuf répondants sur dix ont affirmé qu'une formation aux PS les aurait mis en confiance. En moyenne, 83,5% des répondants ont reconnu ne pas connaitre les gestes de PS à poser dans cinq scenarios (asphyxie secondaire à un corps étranger des voies aériennes, hémorragie externe post-traumatique, convulsion fébrile du nourrisson, obstruction des voies respiratoires chez un adulte inconscient et arrêt cardiaque).

| Tableau 4. Connaissances subjectives concernant les PS | | | | | | |
| --- | --- | --- | --- | --- | --- | --- |
|  | **Zones urbaines Zones périurbaines** | | | | **Total** | |
|  | **n** |  | **n** |  | **n** | **%** |
|  | **1016** | **83.5%** | **201** | **16.5%** | **1217** | **100%** |
| Pense connaitre les gestes des PS pour : | | | | | | |
|  | **1) Enf. 5ans avec étouffement après une fausse route** 1172 100 | | | | | |
| Total | 153 | 85.0 | 27 | 15.0 | 180 | 15.4 |
| Jamais scolarisé | 1 |  | 2 |  | 3 | 1.7 |
| Niveau Primaire + Secondaire | 52 |  | 22 |  | 74 | 41.1 |
| Niveau Tertiaire | 100 |  | 3 |  | 103 | 57.2 |
|  | **2) Enf.12ans avec jambe fracturée saignant abondamment** 1172 100 | | | | | |
| Total | 252 | 91.6 | 23 | 9.4 | 275 | 23.5 |
| Jamais scolarisé | 2 |  | 1 |  | 3 | 1.1 |
| Niveau Primaire + Secondaire | 85 |  | 18 |  | 103 | 37.4 |
| Niveau Tertiaire | 165 |  | 4 |  | 169 | 61.4 |
|  | **3) Adulte en arrêt cardiorespiratoire** 1170 100 | | | | | |
| Total | 111 | 95.5 | 4 | 4.5 | 115 | 9.8 |
| Jamais scolarisé | 0 |  | 0 |  | 0 | 0.0 |
| Niveau Primaire + Secondaire | 30 |  | 2 |  | 32 | 27.8 |
| Niveau Tertiaire | 81 |  | 2 |  | 83 | 72.2 |
|  | **4) Nourrisson de 2 ans avec convulsions fébriles** 1170 100 | | | | | |
| Total | 281 | 92.1 | 24 | 7.9 | 305 | 26.1 |
| Jamais scolarisé | 0 |  | 0 |  | 0 | 0.0 |
| Niveau Primaire + Secondaire | 151 |  | 19 |  | 170 | 55.7 |
| Niveau Tertiaire | 130 |  | 5 |  | 135 | 44.3 |
|  | **5) Adulte inconscient avec respiration sonore** 1171 100 | | | | | |
| Total | 81 | 92.0 | 7 | 8.0 | 88 | 7.5 |
| Jamais scolarisé | 0 |  | 0 |  | 0 | 0.0 |
| Niveau Primaire + Secondaire | 8 |  | 4 |  | 12 | 13.6 |
| Niveau Tertiaire | 73 |  | 3 |  | 76 | 86.3 |
|  |  |  |  |  | 1171 | 100 |
| Moyenne | **175** | **91.0** | **17** | **9.0** | **192** | **16.4** |

**Premiers secours et niveau d'éducation, âge et lieu de résidence**

Alors que les répondants avec un niveau d’études supérieur représentaient 37,6% de notre échantillon, ils ont constitué 64,3% des participants ayant déclaré avoir une connaissance des PS (p<0,01). Ce n'était pas le cas respectivement pour la reconnaissance de l’importance des PS (pour laquelle 35,1% avaient un niveau d’études supérieur) et pour l'attitude positive (pour laquelle les personnes les plus instruites représentaient 37,9%).

Les résidents de ZU ont constitué 91,0% des participants ayant déclaré connaitre les gestes de PS des scenarios (p<0,01) alors qu’ils ne représentaient 83,5% de notre échantillon, ceci probablement en raison de la proportion plus élevée de résidents avec un niveau d’études supérieur en ZU par rapport aux ZPU (41,9% contre 14,9%, p<0,01). Cependant, le lieu de résidence n’a pas semblé être déterminant en ce qui concerne la perception de l’importance des PS (les résidents des ZU représentaient 84,4% alors qu'ils étaient 83,2% de notre échantillon) et l'attitude positive affichée (les résidents des ZU représentaient 77,4%).

Enfin, alors que les répondants âgés de moins de 31ans représentaient 30,9% de notre échantillon, ils ont représenté 25,0% des participants ayant déclaré avoir une connaissance des PS. C'était l'inverse pour les ≥51 ans (21,8 % de l’échantillon et 23,9% de ceux ayant déclaré avoir une connaissance des PS). L'âge était un important discriminateur concernant la perception de l’importance des PS (20,2 % pour les < 31ans et 31,3 % pour les 51ans, p<0,01) et l'attitude favorable (20,8% pour les <31ans et 31,4 % pour les ≥51 ans, p<0,01).

**DISCUSSION**

Cette étude a évalué les perceptions, attitudes et connaissances concernant les PS dans les ménages de Kinshasa. Les 1217 répondants étaient majoritairement des femmes (68,1%), avec un âge moyen était de 39,9 ± 14,4ans, une variabilité qui a permis d'évaluer les possibles différences de points de vue liées à l'âge^[[39]](#endnote-39)^. La majorité des ménages étaient issus d'un milieu socio-économique défavorisé, avec 70,0% vivant avec <100USD par personne par mois. Les répondants des ZU différaient significativement de ceux des ZPU par leur niveau d'éducation plus élevé et un taux de chômage inférieur, ce qui a probablement affecté les revenus moyens des ménages. Nos résultats suggèrent que ces différences ont pu influer sur les réponses dans ces groupes. Comme c'est le cas pour la méconnaissance et le rejet de la vaccination et de la contraception dans les PFR, la pauvreté, le bas niveau d'éducation et l’accès réduit à l’information sont parmi les facteurs qui affectent négativement les vues sur les PS dans les communautés^[[40]](#endnote-40)^.

Les urgences médicales – définies au sens large comme des maladies ou blessures nécessitant des soins de santé dans les minutes ou quelques heures qui suivent – survenaient en effet fréquemment dans les ménages, comme en témoigne le nombre des consultations en urgence enregistrées dans l’enquête (52,6% des ménages), un taux similaire à d'autres études dans les PFR^21 26^. Certains de ces tableaux aigus auraient nécessité des PS à domicile. En revanche, l'utilisation des services d'ambulance était presque inexistante (0,2%), bien en deçà des autres pays africains (Afrique du Sud - 67%, Éthiopie - 20,3% et Ghana - 4,5 %)^37^ ^[[41]](#endnote-41)^ ^[[42]](#endnote-42)^. Toutefois, même dans les pays développés dotés des SAMU efficaces, face aux urgences, les membres du ménage jouent souvent un rôle crucial dans l'administration des PS et l'activation rapide des secours. Ceci fait d’eux un maillon essentiel de la chaîne de soins, quel que soit le milieu^3 4^. Les personnes sans compétence en PS doivent donc se résigner à l’option peu probable de solliciter l'aide d'un voisin (ce qui n'était pas possible dans 63,1% des cas dans notre étude). Sur les 155 décès déclarés dans notre enquête, 20,6% sont survenus en dehors d’une formation sanitaire. Dans ces circonstances, l’administration des PS par un proche était la seule option de soins et aurait probablement pu éviter certains décès. En effet, une revue systématique de la prise en charge des victimes de traumatismes a montré une réduction potentielle de la mortalité lorsque des PS sont administrés avant les soins proprement dits^[[43]](#endnote-43)^.

La majorité des participants (77,6%) ont reconnu qu'une urgence nécessitant des PS pouvait bien se produire un jour dans leur ménage, et 90,0% ont avoué que la connaissance des PS était une nécessité. Ceci est une indication claire de l’importance reconnue aux PS. Bien que notre étude n'ait pas spécifiquement recherché les sources d’information des répondants sur les PS, la différence non significative notée sur ce point entre les résidents des ZPU et ceux des ZU suggérerait un accès réduit aux sources dans le premier groupe. Malgré le fait qu’un grand nombre a reconnu la valeur des PS, le nombre des personnes ayant reçu une formation à Kinshasa était minimal et significativement inférieur à celui d’autres PFR^9 25^ et des pays à revenu élevé ^8 12^. Les causes sont probablement multifactorielles, y compris la rareté des formations offertes, plutôt qu'un manque d'intérêt. Les opportunités de formation sont limitées et sporadiques, principalement organisées par la Croix-Rouge, surtout dans les villes, souvent pour renforcer les capacités des agents de santé communautaires^[[44]](#endnote-44)^; les programmes scolaires officiels tant au niveau primaire que secondaire ne mentionnent pas spécifiquement la formation et pratique des PS comme matière à enseigner^[[45]](#endnote-45)^.

De plus, nos résultats renforcent le besoin vital de formation pour accroitre la pratique de PS à Kinshasa ; l'expérience vécue par certains répondants démontre le danger que représente l’absence de pratique des PS dans les ménages. Indépendamment du lieu de résidence, 47,2% des participants ont déclaré avoir eux-mêmes déjà été directement confrontés dans le passé à une situation d'urgence nécessitant des PS, et 62,1% d'entre eux n’avaient pas confiance en leur capacité à les administrer. Avoir été victime ou témoin d’un accident est cité comme un facteur prédictif pour la reconnaissance de l’importance et de la connaissance des PS^[[46]](#endnote-46)^. Semblable à d’autres études^13 14^ ^[[47]](#endnote-47)^, la grande majorité de nos répondants (90,0%) ont affirmé qu'une formation en PS les aurait rendus plus confiants. En effet, la formation (surtout récente) est corrélée à une connaissance accrue en PS et à l’application effective de ces compétences^[[48]](#endnote-48)^. Même parmi ceux qui n'avaient jamais été confrontés à une urgence nécessitant des PS, 91,3% ont affirmé qu'un cours de formation de base aux PS peut effectivement contribuer à améliorer le pronostic des malades ou victimes^3 4 12^. Cette attitude positive sur les PS a aussi été retrouvée dans plusieurs études similaires^10 25 48^.

En moyenne, 83,5% des participants ont déclaré qu'ils ne pensaient pas connaitre les gestes de PS pour cinq urgences courantes, ce qui est directement corrélé au manque de formation en PS et équivaut à un niveau de connaissance inférieur à celui trouvé dans des études similaires^12 18 25^. En effet, le savoir découle essentiellement de l'apprentissage et de la formation^23^. La majorité des répondants ignoraient les gestes de base des PS tels que les claques dans le dos et les compressions abdominales pour un enfant en étouffement total par suite d’un corps étranger obstruant les voies aériennes supérieures, le contrôle de l'hémorragie par pression directe, l'ouverture des voies respiratoires et la position latérale de sécurité pour un adulte inconscient, et les compressions thoraciques en cas d'arrêt cardiaque. A cet égard, le procédé approprié, objectif et précis pour évaluer le niveau des connaissances théoriques et pratiques demeure une évaluation méthodique formelle. Cependant, l’aveu de manque de connaissance peut être un indicateur indirect^[[49]](#endnote-49)^ ^[[50]](#endnote-50)^. Il est probable que certains parmi les rares qui ont affirmé connaître les gestes de PS pour les 5 scénarios de notre étude ne les maîtrisent pas en réalité. En outre, nos résultats ont révélé une association entre les connaissances subjectives et le niveau d'éducation ; Il y avait plus de participants avec un niveau tertiaire que ceux avec un niveau inférieur parmi les répondants qui avaient déclaré avoir une connaissance des PS, ce qui confirme que le niveau d’éducation est fortement corrélé aux déterminants de la santé et influence les perceptions et les pratiques^18^. De même, certaines études suggèrent aussi un certain degré d'association entre l'âge avancé et une plus grande reconnaissance de l’importance des PS, une attitude positive et une connaissance solide, conformément aux conclusions d'une étude parmi des enseignants en Éthiopie^25^. Ces facteurs méritent d'être étudiés de manière plus approfondie.

**LIMITES**

Cette étude est basée sur les réponses des participants, des données subjectives dont l'exactitude n’a pu être confirmée indépendamment. De plus, certains participants peuvent avoir eu un biais de mémoire ou avoir fourni des réponses socialement acceptables. En outre, des termes comme « urgences médicales », « premiers secours » ou « connaissance » utilisés dans le questionnaire ont des sens larges et, malgré les efforts des enquêteurs pour expliquer clairement leurs significations, ils peuvent avoir été compris diversement. Enfin, le moyen plus précis d'évaluer les connaissances sur les PS aurait été de les tester de manière objective et pratique au lieu des avis subjectifs. Cependant, cette étude fondamentale offre une base à partir de laquelle des projets de recherche plus élaborés sur les PS peuvent être menés dans le futur.

**CONCLUSION**

La connaissance des premiers secours est un atout majeur pour chaque adulte et grand enfant. Malgré une reconnaissance de leur importance et une attitude positive à leur égard, il y a actuellement une grande méconnaissance des premiers secours dans les ménages de Kinshasa. Des programmes de formation adaptés au contexte local sont indispensables pour doter les communautés des compétences de base pour préserver la vie, soulager la souffrance et améliorer la riposte face aux urgences. Dans le cadre des efforts pour le développement du système des soins préhospitaliers en RDC, il est essentiel de disponibiliser des formations et programmes éducatifs appropriés pour le grand public de Kinshasa.

**REFERENCES**

1. Reynolds T, Sawe H, Rubiano A, et al. Strengthening Health Systems to Provide Emergency Care. Disease Control Priorities: Improving Health and Reducing Poverty. 3 ed. Washington (DC): The International Bank for Reconstruction and Development / The World Bank 2017:247-65. [↑](#endnote-ref-1)
2. Zideman D, Singletary E, Borra V et al.: European Resuscitation Council Guidelines 2021: First aid. Resuscitation, Vol 161, p270-290, April 2021. <https://doi.org/10.1016/j.resuscitation.2021.02.013> [↑](#endnote-ref-2)
3. International Red Cross and Red Crescent Societies / First aid for a safer future: updated global edition / September 2010. Available online and accessed on Aug 08 2021: <https://www.globalfirstaidcentre.org/wp-content/uploads/2020/11/First-aid-for-a-safer-future-Updated-global-edition-Advocacy-report-2010-2.pdf/> [↑](#endnote-ref-3)
4. Singletary E, Zideman D, Bendall J et al.: 2020 International Consensus on First Aid Science with Treatment Recommendations. Circulation. 2020;142(suppl 1): p284–p334.

   DOI: 10.1161/CIR.0000000000000897 Downloaded at <https://www.ahajournals.org/journal/circ> [↑](#endnote-ref-4)
5. Balikuddembe, J.K., Ardalan, A., Khorasani-Zavareh, D. *et al.* Weaknesses and capacities affecting the Prehospital emergency care for victims of road traffic incidents in the greater Kampala metropolitan area: a cross-sectional study. *BMC Emerg Med* 17**,**29 (2017). <https://doi.org/10.1186/s12873-017-0137-2> [↑](#endnote-ref-5)
6. Obermeyer Z, Abujaber S, Makar M, et al. Emergency care in 59 low- and middle-income countries: a systematic review. *Bulletin of the World Health Organization* 2015;93(8):577-86G. doi: 10.2471/BLT.14.148338 [published Online First: 2015/10/20] [↑](#endnote-ref-6)
7. Mould-Millman NK DJ, Sefa N, Yancey A, Hollong BG, Hagahmed M, Ginde AA, Wallis LA. The State of Emergency Medical Services (EMS) Systems in Africa. *Prehosp Disaster Med* 2017;32(3):1-12. [↑](#endnote-ref-7)
8. Wissenberg M, Lippert F, Folke F et al.: Association of National Initiatives to Improve Cardiac Arrest Management with Rates of Bystander Intervention and Patient Survival After Out-of-Hospital Cardiac Arrest. ,*JAMA*. 2013;310(13):1377-1384. doi:10.1001/jama.2013.278483 [↑](#endnote-ref-8)
9. Olugbenga-Bello A, Adefisoye A et al.: First aid knowledge and application among commercial inter-city drivers in Nigeria. African Journal of Emergency Medicine (2012) 2, 108–113 <http://dx.doi.org/10.1016/j.afjem.2012.06.003> [↑](#endnote-ref-9)
10. Hoque D, Islam I, Salam S, Alonge O et al.: Impact of First Aid on Treatment Outcomes for Non-Fatal Injuries in Rural Bangladesh: Findings from an Injury and Demographic Census. Int. J. Environ. Res. Public Health 2017, 14, 762; doi:10.3390/ijerph14070762 [↑](#endnote-ref-10)
11. [Tannvik](https://www.researchgate.net/scientific-contributions/T-D-Tannvik-84550232?_sg%5B0%5D=-qYHzswCk1JJrBKvXSwLNZWcVb0Q31Rcv5VcrlE8_czfYyic5X4AH5pgt7YT_uw1q0Km6t0.qmuV_RQMPFiqXPkOSWAi3f3U_UNMLSvYkYwKy4HZSxrfY0QwyEmZB3BnsGhX8fIByjepl_0BLXWCrVRsxGys3w&_sg%5B1%5D=G-PLQsUD_wCSIFkbqsFhsvSh-Cd9j2Ali18pfCHfJNtwf1hMcZdIeHGpvombmietZfGRTIo.FJeCBKd-lDdfqf10FsPyh1xDiHHZ3Krf2xq1ZsZnVI6_JyhL8joi7ihIldqDfH_f_lf8V60t45JGEOkw82rszA) TD, Bakke HK, Wisborg T. A systematic literature review on first aid provided by laypeople to trauma victims. 2012. Acta Anaesthesiologica Scandinavica 56(10):1222-7. DOI: [10.1111/j.1399-6576.2012.02739.x](http://dx.doi.org/10.1111/j.1399-6576.2012.02739.x) [↑](#endnote-ref-11)
12. Midani O, Tillawi T, Saqer A, Hammami MB, Taifour H, Mohammad H. Knowledge and attitude toward first aid: A cross-sectional study in the United Arab Emirates. Avicenna J Med 2018. DOI: 10.4103/AJM.AJM_140_18 [↑](#endnote-ref-12)
13. Pawłowski W, Lasota D, Goniewicz K, Goniewicz M. Effects of first aid training in emergency preparedness and response. Medical Studies/Studia Medyczne 2018; 34 (3): 259–263. DOI: <https://doi.org/10.5114/ms.2018.78691>. [Accessed Aug 19 2021]. [↑](#endnote-ref-13)
14. Ahmed WAM, Salman AO, Arafa KA. Households’ preparedness for first-aid of burns and falls in Khartoum.2014. African Journal of Emergency Medicine (2014) 4, 184–187.

    DOI: http://dx.doi.org/10.1016/j.afjem.2014.07.010 [↑](#endnote-ref-14)
15. Oliver E, Cooper J, McKinney D. Can first aid training encourage individuals’ propensity to act in an emergency situation? A pilot study. 2012. EMJ Online First, published on June 28, 2013. doi:10.1136/emermed-2012-202191 [↑](#endnote-ref-15)
16. Yan S, Gan Y, Chuanzhu L et al. The global survival rate among adult out-of-hospital cardiac arrest patients who received cardiopulmonary resuscitation: a systematic review and meta-analysis. Critical Care (2020) 24:61. <https://doi.org/10.1186/s13054-020-2773-2> [↑](#endnote-ref-16)
17. International Federation of Red Cross and Red Crescent Societies International first aid and resuscitation guidelines 2016. Available at <https://www.ifrc.org/Global/Publications/Health/First-Aid-2016-Guidelines_EN.pdf>. Accessed on 17 Aug 2021 [↑](#endnote-ref-17)
18. O’Donnell O et al. 1 Access to health care in developing countries: breaking down demand side barriers. Cad. Saúde Pública 23 (12). 2007.  <https://doi.org/10.1590/S0102-311X2007001200003> [↑](#endnote-ref-18)
19. David DH Peters, Garg A, Bloom G, Walker D, Brieger W, Rahman MH. Poverty and Access to Health Care in Developing Countries. 2008. Annals of the New York Academy of Sciences, 1136(1):161 - 71DOI: 10.1196/annals.1425.011 [↑](#endnote-ref-19)
20. H. Gafoor K A. Considerations in measurement of awareness. [National Level Seminar on Emerging Trends in Education. 2012. Available at https://files.eric.ed.gov/fulltext/ED545374.pdf Accessed online on 16.09.2021](C:\\Users\\cmo\\Desktop\\PhD\\Study 5 articles\\For publication\\National Level Seminar on Emerging Trends in Education. 2012. Available at https:\\files.eric.ed.gov\\fulltext\\ED545374.pdf Accessed online on 16.09.2021) [↑](#endnote-ref-20)
21. [Chirongoma](https://www.ncbi.nlm.nih.gov/pubmed/?term=Chirongoma%20F%5BAuthor%5D&cauthor=true&cauthor_uid=28955424) F, [Chengetanai](https://www.ncbi.nlm.nih.gov/pubmed/?term=Chengetanai%20S%5BAuthor%5D&cauthor=true&cauthor_uid=28955424) S, [Tadyanemhandu](https://www.ncbi.nlm.nih.gov/pubmed/?term=Tadyanemhandu%20C%5BAuthor%5D&cauthor=true&cauthor_uid=28955424) C. First aid practices, beliefs, and sources of information among caregivers regarding paediatric burn injuries in Harare, Zimbabwe: A cross-sectional study. Malawi Medical Journal 29 (2): June 2017 [↑](#endnote-ref-21)
22. Visser PS et al. Attitudes. Corsini encyclopedia of psychology. 2010. Vol. 1. 4th ed. 182–84. New York: Wiley. DOI: 10.1002/9780470479216 [↑](#endnote-ref-22)
23. National Academies Press. How People Learn II: Learners, Contexts, and Cultures.

    DOI 10.17226/24783. Available at <http://nap.edu/24783> Accessed on 21 Sept 201. [↑](#endnote-ref-23)
24. Bottiger BW, Lockey A et al. All citizens of the world can save a life”. Resuscitation. VOLUME 128, P188-190. DOI: <https://doi.org/10.1016/j.resuscitation.2018.04.015> [↑](#endnote-ref-24)
25. Ganfure G, Ameya G, Tamirat A, Lencha B, Bikila D. First aid knowledge, attitude, practice, and associated factors among kindergarten teachers of Lideta sub-city Addis Ababa, Ethiopia. PLoS ONE 13(3): e0194263. 2018. <https://doi.org/10.1371/journal.pone.0194263> [↑](#endnote-ref-25)
26. Workneh et al. Determinants of knowledge, attitude, and practice towards first aid among

    kindergarten and elementary school teachers in Gondar city, Northwest Ethiopia. BMC Emergency Medicine (2021) 21:73 <https://doi.org/10.1186/s12873-021-00468->6 [↑](#endnote-ref-26)
27. Chokotho L, Mulwafu W, Singini I , Njalale Y , MaliwichiSenganimalunje L , Jacobsen KH . First Responders and Prehospital Care for Road Traffic Injuries in Malawi. *Prehosp Disaster Med*. 2017;32(1):14–19. DOI: <https://doi.org/10.1017/S1049023X16001175> [↑](#endnote-ref-27)
28. Evans D. Evaluating the need for first aid and basic life support training among early childhood development practitioners in Cape Town, South Africa. 2015. UCT Open Library. Accessed 16-8-2021 <https://open.uct.ac.za/bitstream/handle/11427/19884/thesis_hsf_2015_evans_derrick_reginald.pdf?sequence=1> [↑](#endnote-ref-28)
29. Gyedu, A., Stewart, B., Otupiri, E., Donkor, P., & Mock, C. (2021). First Aid Practices for Injured Children in Rural Ghana: A Cluster-Random Population-Based Survey. *Prehospital and Disaster Medicine,* *36*(1), 79-85. doi:10.1017/S1049023X20001430 [↑](#endnote-ref-29)
30. Democratic Republic of the Congo Geneva: World Health Organization; 201 [Available from: [https://www.who.int/countries/cod/29 August 2021](https://www.who.int/countries/cod/29%20August%202021) [↑](#endnote-ref-30)
31. Malemo LK, Salmon M, Manwa K, Mundenga M, Diango K, Zaidi R, Wendel R, Reynolds TA. The state of emergency care in Democratic Republic of Congo. African Journal of Emergency Medicine, Volume 5, Issue 4. 2015,153-58,

    <https://doi.org/10.1016/j.afjem.2015.08.001> [↑](#endnote-ref-31)
32. Stein C, Mould-Millman NK, De Vries S, Wallis L. Access to out-of-hospital emergency care in Africa: Consensus conference recommendations. African Journal of Emergency Medicine, Vol 6, Issue 3. 2016. 158-61, DOI: <https://doi.org/10.1016/j.afjem.2016.08.008> [↑](#endnote-ref-32)
33. Banque Mondiale. Revue de l’urbanisation en République Démocratique du Congo: des villes productives et inclusives pour l’émergence de la République Démocratique du Congo. Directions Du Développement. Washington, DC: La Banque mondiale; 2018. doi:10.1596/978-1-4648-1205-7. Available at https://openknowledge.worldbank.org/bitstream/handle/10986/28931/9781464812057.pdf?sequence=4 [Accessed on 30 September 2021] [↑](#endnote-ref-33)
34. United Nations Data. DRC Population. 2021. Available at <https://data.un.org/CountryProfile.aspx/_Images/CountryProfile.aspx?crName=Democratic%20Republic%20of%20the%20Congo> [Accessed in 19 June 2021] [↑](#endnote-ref-34)
35. Ministère du Plan et Suivi de la Mise en œuvre de la Révolution de la Modernité - MPSMRM/Congo, Ministère de la Santé Publique - MSP/Congo and ICF International Enquête Démographique et de Santé en République Démocratique du Congo 2013-2014. Rockville, Maryland, USA: MPSMRM, MSP, and ICF International; 2014. Available at <https://dhsprogram.com/publications/publication-fr300-dhs-final-reports.cfm>

    [Accessed on 23 Sept 2021]. [↑](#endnote-ref-35)
36. Ro YS, Shin SD, Jeong J et al. Evaluation of demands, usage and unmet needs for emergency care in Yaounde, Cameroon: A cross-sectional study. BMJ Open 2017;7(2):e014573. DOI: 10.1136/bmjopen-2016-014573 [↑](#endnote-ref-36)
37. Hodkinson PW, Pigoga JL, Wallis L. Emergency healthcare needs in the Lavender Hill suburb of Cape Town, South Africa: a cross-sectional community-based household survey. BMJ Open 2020;10(1):e033643. DOI: 10.1136/bmjopen-2019-033643 [↑](#endnote-ref-37)
38. Application of SurveyCTO mobile data collection technology in household surveys: The case of an impact evaluation of the Community Based Integrated Water Resource Management Project in Niger. 2020.Available at <https://www.researchgate.net/publication/344073313> [↑](#endnote-ref-38)
39. Neis MB [↑](#endnote-ref-39)
40. Zajacova A [↑](#endnote-ref-40)
41. Sultan M et al. Trends and barriers of emergency medical service use in Addis Ababa; Ethiopia. BMC Emergency Medicine (2019) 19:28 <https://doi.org/10.1186/s12873-019-0242-5> [↑](#endnote-ref-41)
42. Mould-Millman NK, Rominski S, Bogus J, Ginde AA, Zakariah A, Boatemaah C, Yancey C, Akoriyea S, Campbell T. Barriers to Accessing Emergency Medical Services in Accra, Ghana: Development of a Survey Instrument and Initial Application in Ghana. Global Health: Science and Practice. 2015, 3 (4) 577-90; DOI: 10.9745/GHSP-D-15-00170 [↑](#endnote-ref-42)
43. Tannvik TD, Bakke HK, Wisborg T. A systematic literature review on first aid provided by

    laypeople to trauma victims. Acta Anaesthesiologica Scandinavica. 2012; ***56:*** *1222–27*

    doi: 10.1111/j.1399-6576.2012.02739.x [↑](#endnote-ref-43)
44. Rapport Annuel 2020 de la Croix-Rouge de la RD Congo. Available online at

    <https://data-api.ifrc.org/documents/CD/AR_CongoTheDemocraticRepublicof_2020.pdf>

    Accessed on 13 August 2021 [↑](#endnote-ref-44)
45. Programme National de l’Enseignement Primaire. Ministère de l’Enseignement Primaire, Secondaire et Professionnel de la République Démocratique du Congo. 2011. Available online at <https://www.eduquepsp.education/sgc/wp-content/uploads/2018/07/Programme_national_primaire_v_2011.pdf>

    Accessed on 13 Aug 2021 [↑](#endnote-ref-45)
46. Huang, L.C.1; Wei, Y.L.2; Chen, L.L.3; Peng, N.H.4 ABSTRACT 790, Pediatric Critical Care Medicine: May 2014 - Volume 15 - Issue 4_suppl - p 177

    doi: 10.1097/01.pcc.0000449516.53685.be [↑](#endnote-ref-46)
47. Abelsson, A., Odestrand, P. & Nygårdh, A. To strengthen self-confidence as a step in improving prehospital youth laymen basic life support. *BMC Emerg Med* 20, 8 (2020). <https://doi.org/10.1186/s12873-020-0304-8> [↑](#endnote-ref-47)
48. Kano M, Siegel JM, Bourque LB. First-aid training and capabilities of the lay public: a potential alternative source of emergency medical assistance following a natural disaster. Disasters. 2005;29(1):58–74. [PubMed: 15720381] [↑](#endnote-ref-48)
49. Z. Hansson I, Buratti S and Allwood CM. Experts’ and Novices’ Perception of Ignorance and Knowledge in Different Research Disciplines and Its Relation to Belief in Certainty of Knowledge. 2017. Front. Psychol. 8:377. doi: 10.3389/fpsyg.2017.00377 [↑](#endnote-ref-49)
50. Park CW, Gardner MP, Thukral VK. Self-Perceived Knowledge: Some Effects on Information Processing for a Choice Task.The American Journal of Psychology 101, no. 3 (1988): 401–24. https://doi.org/10.2307/1423087. [↑](#endnote-ref-50)
